# Plasma Gelsolin Enhances Phagocytosis of Candida auris by Human Neutrophils through Scavenger Receptor Class B

**DOI:** 10.1128/spectrum.04082-22

**Published:** 2023-02-21

**Authors:** Łukasz Suprewicz, Karol Skłodowski, Alicja Walewska, Piotr Deptuła, Alicja Sadzyńska, Andrzej Eljaszewicz, Marcin Moniuszko, Paul A. Janmey, Robert Bucki

**Affiliations:** a Department of Medical Microbiology and Biomedical Engineering, Medical University of Białystok, Białystok, Poland; b Department of Regenerative Medicine and Immune Regulation, Medical University of Białystok, Białystok, Poland; c Prof. Edward F. Szczepanik State Vocational University—Suwałki, Suwałki, Poland; d Department of Physiology, University of Pennsylvania, Philadelphia, Pennsylvania, USA; e Institute for Medicine and Engineering, University of Pennsylvania, Philadelphia, Pennsylvania, USA; Geisel School of Medicine at Dartmouth

**Keywords:** *Candida auris*, inflammation, neutrophils, phagocytosis, plasma gelsolin

## Abstract

In addition to its role as an actin-depolymerizing factor in the blood, plasma gelsolin (pGSN) binds bacterial molecules and stimulates the phagocytosis of bacteria by macrophages. Here, using an *in vitro* system, we assessed whether pGSN could also stimulate phagocytosis of the fungal pathogen Candida auris by human neutrophils. The extraordinary ability of C. auris to evade immune responses makes it particularly challenging to eradicate in immunocompromised patients. We demonstrate that pGSN significantly enhances C. auris uptake and intracellular killing. Stimulation of phagocytosis was accompanied by decreased neutrophil extracellular trap (NET) formation and reduced secretion of proinflammatory cytokines. Gene expression studies revealed pGSN-dependent upregulation of scavenger receptor class B (SR-B). Inhibition of SR-B using sulfosuccinimidyl oleate (SSO) and block lipid transport-1 (BLT-1) decreased the ability of pGSN to enhance phagocytosis, indicating that pGSN potentiates the immune response through an SR-B-dependent pathway. These results suggest that the response of the host’s immune system during C. auris infection may be enhanced by the administration of recombinant pGSN.

**IMPORTANCE** The incidence of life-threatening multidrug-resistant Candida auris infections is rapidly growing, causing substantial economic costs due to outbreaks in hospital wards. Primary and secondary immunodeficiencies in susceptible individuals, such as those with leukemia, solid organ transplants, diabetes, and ongoing chemotherapy, often correlate with decreased plasma gelsolin concentration (hypogelsolinemia) and impairment of innate immune responses due to severe leukopenia. Immunocompromised patients are predisposed to superficial and invasive fungal infections. Morbidity caused by C. auris among immunocompromised patients can be as great as 60%. In the era of ever-growing fungal resistance in an aging society, it is critical to seek novel immunotherapies that may help combat these infections. The results reported here suggest the possibility of using pGSN as an immunomodulator of the immune response by neutrophils during C. auris infection.

## INTRODUCTION

Plasma gelsolin (pGSN) is an extracellular isoform of the protein gelsolin expressed in most human cells, classified as a Ca^2+^/phosphatidylinositol 4,5-bisphosphate-regulated actin-binding protein (ABP) (1). pGSN, apart from being the primary component of the actin scavenger system in the blood, is a protein with pleiotropic functions that can bind products of bacterial origin, such as lipopolysaccharide (LPS) and lipoteichoic acid (LTA) (2, 3). Under physiological conditions, the concentration of pGSN in human blood varies between 150 and 300 μg/mL (4, 5). Hypogelsolinemia, a decreased plasma gelsolin concentration, is noted when circulating pGSN is consumed in severe infectious and noninfectious conditions, such as sepsis and septic shock, major trauma, and tissue injury (6–9). Importantly, hypogelsolinemia correlates with poor clinical outcomes (10). Recent reports indicate that pGSN stimulates phagocytosis in mice with sepsis caused by Pseudomonas aeruginosa infection (11). pGSN was reported to be a key factor modulating host immune responses due to the preferential binding of microbially derived endotoxins, resulting in the prevention of toll-like receptor (TLR) activation (2, 3). However, little is known about the mechanisms and ability of pGSN to stimulate innate immune responses independent of direct binding to microbial products. In our work, we show that the immunomodulatory properties of pGSN depend in part on the stimulation of scavenger receptor class B (SR-B) expression on the surface of human neutrophils. SR-B is a group of transmembrane glycoproteins comprised of three members, SR-B1 (SCARB1), SR-B2 (CD36), and SR-B3 (SCARB2), known to bind and internalize a broad variety of ligands (12). SR-B expressed in neutrophils and macrophages plays a role in innate immunity by binding pathogen-associated molecular patterns (PAMPs) or damage-associated molecular patterns (DAMPs) and internalizing pathogens (13).

Candida auris is an emerging multidrug-resistant yeast that causes severe invasive infections with a mortality rate of up to 60% (14). It was first discovered in Japan in 2009, and since then, individual cases or outbreaks have been reported from over 20 countries on six continents (15, 16). Controlling C. auris is challenging since it is resistant to multiple classes of antifungals, can be misidentified as other yeasts by commonly available identification methods, and is able to colonize patients, perhaps indefinitely, and persist in the health care environment, where it can spread between patients (17, 18). The transmissibility, ability to evade innate immune responses, and high levels of antifungal resistance characteristic of C. auris set it apart from most other *Candida* species (19, 20). In the past decade, fungal infections have become a severe medical concern in hemato-oncology, transplantology, geriatric, pulmonology, and intensive care unit hospital wards (21, 22). Statistically, the incidence of life-threatening fungal infections is steadily growing, generating substantial economic costs (23). Among the most important reasons for this increase are an aging society, a growing number of chronic medical conditions affecting patients, and selection for drug-resistant pathogens (24). Primary and secondary immunodeficiencies in susceptible individuals, such as those with leukemia, solid organ transplants, diabetes, and ongoing chemotherapy, are considered the main risk factors that predispose them to superficial and invasive fungal infections, resulting in high morbidity and mortality (25). In contrast to antibiotics, the number of agents with potent antifungal activity is significantly limited, and those approved for clinical use are often very toxic or insufficiently effective, which motivates research and development of agents with novel and alternative mechanisms of action (26).

In this study, we examine the role of pGSN in the innate immune response of human neutrophils during C. auris infection *in vitro*. We evaluated the phagocytic efficacy of neutrophils using various yeast inocula. In addition, we assessed the formation of extracellular neutrophil traps (NETs) and the secretion of inflammatory mediators. Finally, the expression of genes involved in phagocytosis was screened to determine the mechanism of action of pGSN in the immune response, which was further confirmed with antagonists of selected targets.

## RESULTS

### Plasma gelsolin improves the phagocytic efficacy of human neutrophils.

**FIG 1:**
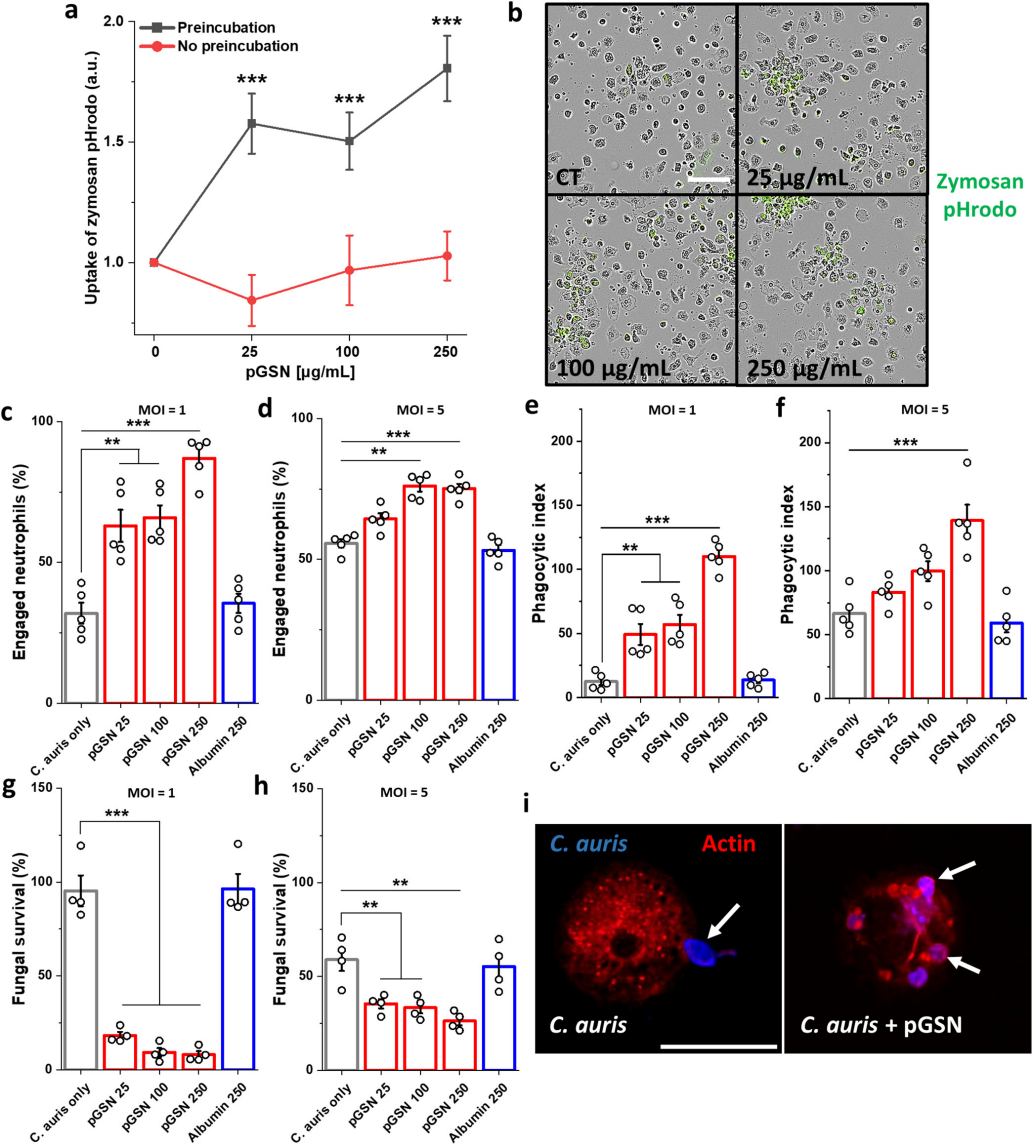
Plasma gelsolin enhances uptake and intracellular killing of C. auris cells by human neutrophils. (a) Live cell imaging data of the uptake of zymosan pHrodo particles after 2 h by human neutrophils that were serum starved for 1 h and either preincubated with pGSN for 1 h or had pGSN simultaneously added with the zymosan particles (*n* = 4). Results were normalized to the untreated control (0), set as 1. (b) Representative images of the uptake of zymosan particles (green) by pGSN-pretreated neutrophils; bar, ~100 μm. (c to f) Percentage uptake and phagocytic index for pGSN-preincubated human neutrophils ingesting C. auris cells (*n* = 5) at MOIs of 1 (c and e) and 5 (d and f). Neutrophils taking up at least one fungal cell were manually tracked to allow a quantitative analysis of percentage uptake during the 2-h coincubation period (c and d). The number of fungal cells ingested (phagocytic index) by the neutrophils was manually counted after 2 h of coincubation (e and f). The survival of C. auris cells after infection of pGSN-pretreated (1 h) human neutrophils (1 h serum starved) at MOIs of 1 (g) and 5 (h) for 2 h was evaluated by plating dilutions of samples on Sabouraud agar (*n* = 4). (i) Images of neutrophils that were serum starved, pretreated or not with pGSN, cocultured with Calcofluor white (blue)-stained C. auris cells for 2 h, fixed, and immunolabeled for actin with Texas red phalloidin (red). White arrows indicate C. auris cells. Bar, ~15 μm. Data are presented as the mean ± standard error of the mean (SEM). **, *P* < 0.01; ***, *P* < 0.001. Significance was determined by Student’s *t* test (a) or one-way analysis of variance (ANOVA) with Tukey’s test (c to h). Human albumin was used as a negative control.

Neutrophils are the first line of defense against both bacterial and fungal infections. Phagocytosis, a nonspecific cellular process for ingesting and eliminating particles larger than 0.5 μm in diameter, including microorganisms such as fungi, is a primary mechanism exploited by neutrophils in innate defense (27). To assess whether and how pGSN stimulates phagocytosis, we performed an uptake assay of zymosan particles by human neutrophils (Fig. 1a and b). To accomplish this, neutrophils were serum starved for 1 h to prevent the potential effect of pGSN present in the blood serum and then were preincubated with pGSN (1 h). Alternatively, pGSN was added simultaneously with zymosan molecules (2 h). As previously reported (11), pGSN stimulates phagocytosis when preincubated with cells for 1 h before their exposure to zymosan, but we did not observe an increase in zymosan uptake when pGSN was added simultaneously with the particles, suggesting that its immunomodulatory effects require priming of the neutrophils.

In the next stage of the study, neutrophils were serum starved for 1 h, preincubated with pGSN (1 h), and infected with C. auris (2 h) to assess whether pGSN would also stimulate phagocytosis in this setting. Evasion of the immune response is characteristic of C. auris, and its severity is strain dependent (19, 28, 29). To rule out a nonspecific protein effect of pGSN, we used human albumin as a control. Previous reports indicated that the amount of C. auris in infected tissues remained high regardless of the inoculum used (28). For this reason, we used fungal loads at a multiplicity of infection (MOI; yeasts to neutrophils) of 1 and 5 to assess whether pGSN would improve neutrophil phagocytic efficiency irrespective of the inoculum. We observed a significant increase in the phagocytic efficacy of the human neutrophils during C. auris infection upon pGSN preincubation. The number of neutrophils involved in phagocytosis, i.e., those with at least one fungal cell in their cytoplasm or adherent to them, increased significantly upon preincubation with pGSN (Fig. 1c and d). Similarly, we observed an increase in the phagocytosis index after preincubation with pGSN, which was very low under the control conditions (Fig. 1e and f), confirming the previously reported evasion of innate immunity by C. auris. We also observed a substantial increase in C. auris eradication after the addition of pGSN (Fig. 1g and h).

### Inhibition of NETotic death of human neutrophils.

When exposed to harmful substances, neutrophils can secrete the contents of their cytoplasm in the process of NETosis, a distinct form of active cell death characterized by releasing decondensed chromatin and granular contents into the extracellular space (30). Despite its essential role in fighting infection, NETosis can be a double-edged sword. Excessive formation of neutrophil extracellular traps (NETs) can lead to vascular damage and increased inflammation due to cytokine and reactive oxygen species (ROS) oversecretion (31).

**FIG 2:**
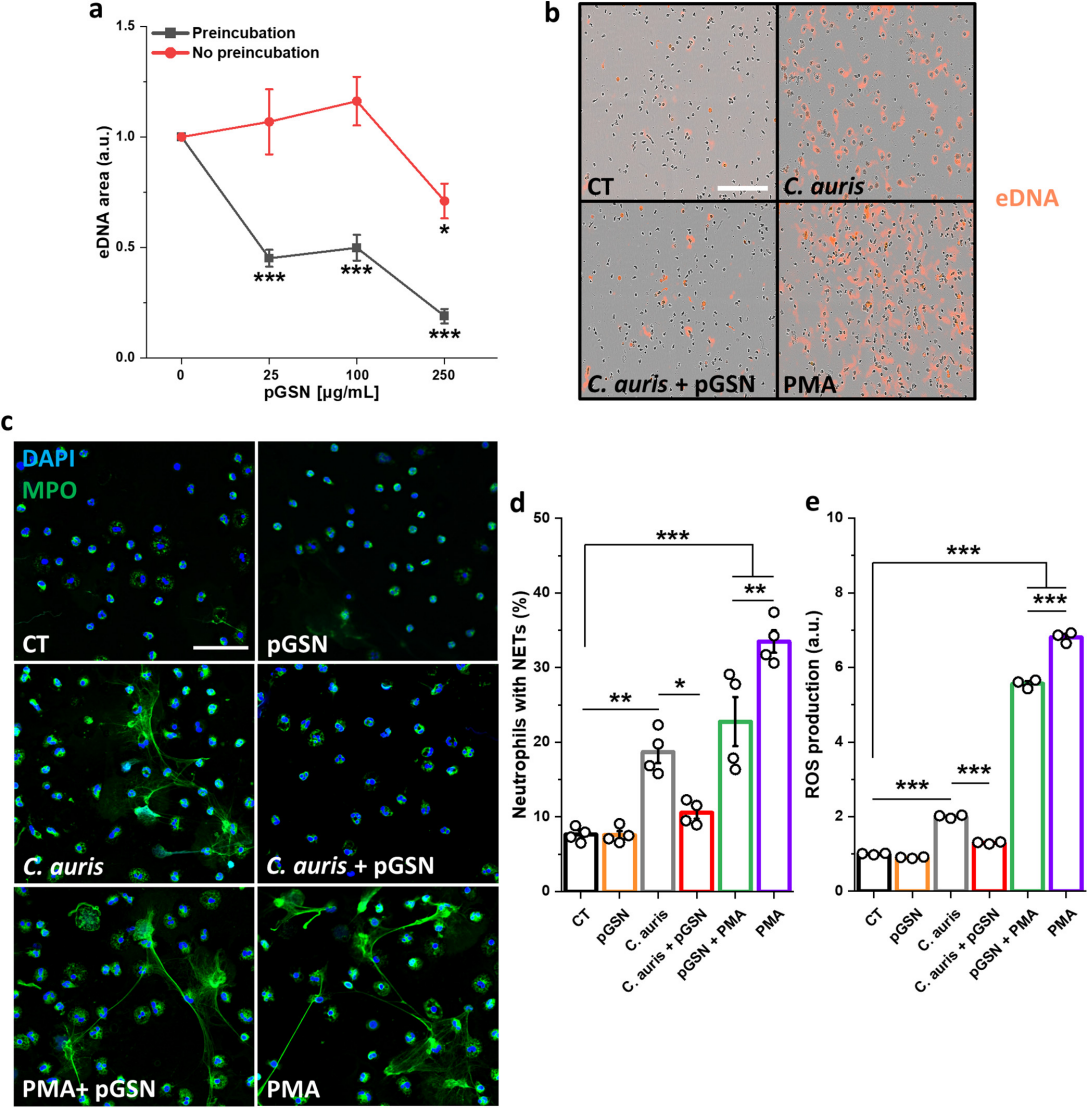
Plasma gelsolin prevents NETotic death of human neutrophils upon C. auris infection. (a) Live cell imaging data of extracellular DNA release (eDNA) by human neutrophils that were either preincubated with pGSN for 1 h or had pGSN simultaneously added to them with the C. auris cells at an MOI of 5 for 2 h (*n* = 4). (b) Representative images of pGSN-pretreated neutrophils releasing eDNA (orange) upon C. auris infection. Bar, ~250 μm. (c) Images of NETs released by human neutrophils that were pretreated as previously described; myeloperoxidase (MPO) is shown in green, while blue indicates DNA stained with DAPI. Bar, ~50 μm. (d) Percentage of NETs (*n* = 4) and (e) release of the reactive oxygen species (*n* = 3) from human neutrophils (pGSN pretreated, as previously) upon C. auris infection at an MOI of 5. Data are presented as the mean ± SEM. Results were normalized to the untreated control (0 or CT), set as 1 (a and e). *, *P* < 0.05; **, *P* < 0.01; ***, *P* < 0.001. Significance was determined by Student’s *t* test (a) or one-way ANOVA with Tukey’s test (d and e). PMA (phorbol-12-myristate-13-acetate) was used as a positive control.

**FIG 3:**
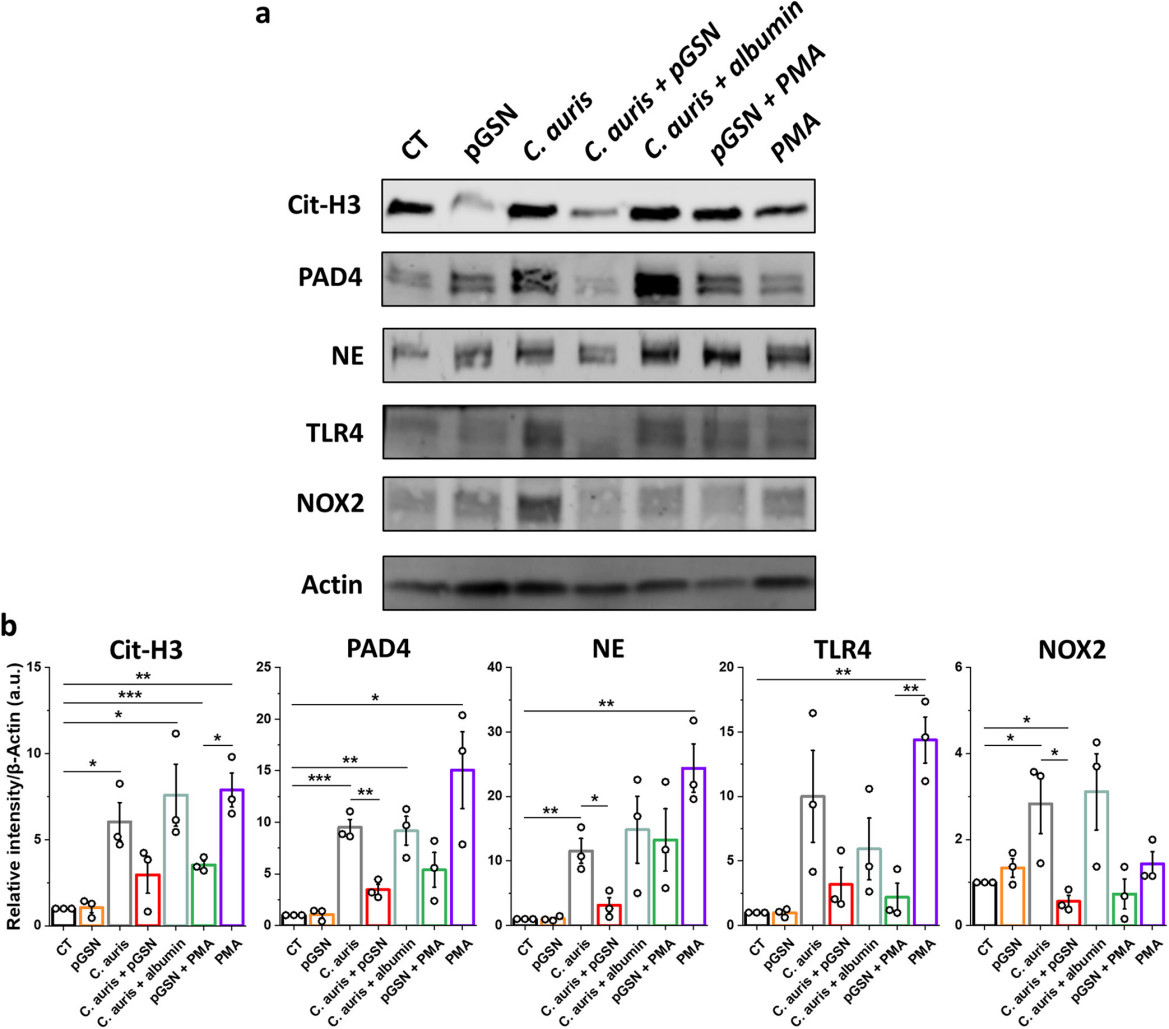
Plasma gelsolin inhibits the expression of proteins involved in NET formation. (a) Immunoblot analysis for the indicated proteins using lysates from human neutrophils that were serum starved for 1 h, then preincubated with pGSN at 250 μg/mL for 1 h, washed, and finally infected with live C. auris yeast cells (MOI = 5) for 2 h. Data are expressed as the mean ± SEM and represent three independent experiments. Results were normalized to the expression of the β-actin and are presented relative to those of the negative control (CT), set as 1. *, *P* < 0.05; **, *P* < 0.01; ***, *P* < 0.001. Significance was determined by Student’s *t* test (b). PMA (phorbol-12-myristate-13-acetate) and human albumin were used as the positive and negative controls, respectively.

To evaluate how pGSN influences the formation of NETs, neutrophils were serum starved (1 h) and preincubated with pGSN (1 h); alternatively, pGSN was added simultaneously with C. auris cells (2 h) at an MOI of 5. We observed a significant decrease in extracellular DNA release from human neutrophils upon the addition of pGSN (Fig. 2a and b). Additionally, fluorescence staining of myeloperoxidase (MPO) during C. auris infection was performed (Fig. 2c). A decrease in the number of neutrophils forming NETs and production of ROS was observed when neutrophils were preincubated with pGSN and then infected with C. auris cells. The fact that inhibition of NET formation required preincubation of the neutrophils with pGSN before the addition of C. auris cells (Fig. 2a) suggests that the target of pGSN is the neutrophil rather than C. auris or the exosomes it produces (32). Moreover, we also observed that pGSN inhibited the action of PMA (phorbol 12-myristate 13-acetate), suggesting a robust anti-inflammatory effect of pGSN (Fig. 2d and e). To further confirm these findings, Western blotting was performed to determine the expression of key proteins involved in NET formation (Fig. 3a) (33). We observed a significant increase in the expression of Cit-H3, PAD4, NE, TLR4, and NOX2 caused by the C. auris strain used in our study. The increase in expression of those proteins was significantly inhibited when neutrophils were preincubated with pGSN prior to infection (Fig. 3b).

### Plasma gelsolin attenuates inflammation caused by C. auris.

**FIG 4:**
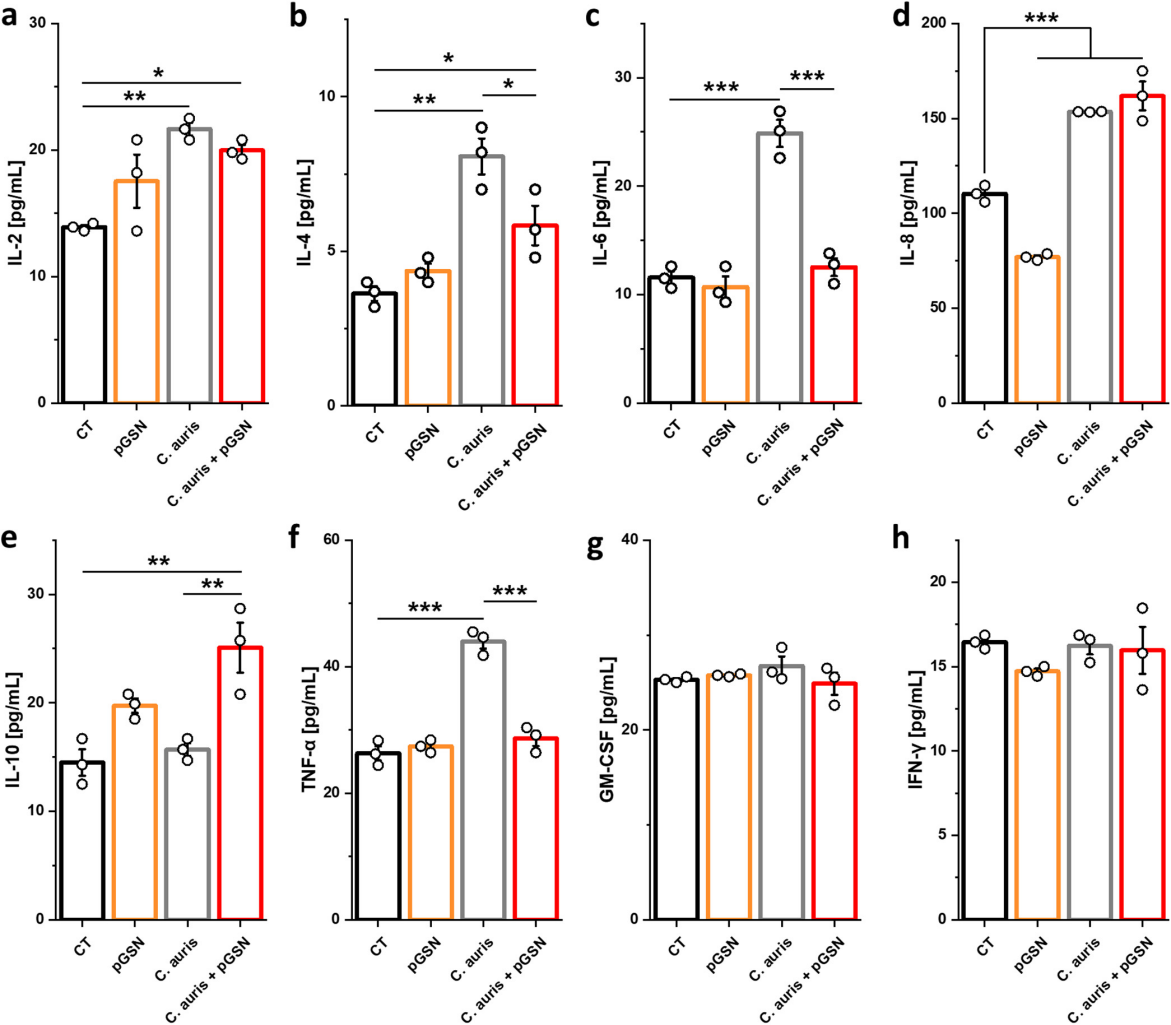
Plasma gelsolin triggers an anti-inflammatory phenotype in human neutrophils during C. auris infection. Production of IL-2 (a), IL-4 (b), IL-6 (c), IL-8 (d), IL-10 (e), TNF-α (f), GM-CSF (g), and IFN-γ (h), as determined by magnetic bead-based enzyme-linked immunosorbent assay (ELISA), in the culture supernatants of human neutrophils that were serum starved for 1 h, preincubated with pGSN at 250 μg/mL for 1h, washed, and infected with live C. auris yeast cells (MOI = 5) for 2 h (*n* = 3). Data are expressed as the mean ± SEM. *, *P* < 0.05; **, *P* < 0.01; ***, *P* < 0.001. Significance was determined by one-way ANOVA with Tukey’s test.

Acute inflammation usually occurs immediately after infection, which will cause the secretion of soluble mediators like cytokines, acute-phase proteins, and chemokines to promote the migration of immune cells to the area of inflammation (34). Although the inflammatory response is a vital process, its prolongation can lead to severe tissue injury, septic shock, and death (35). To evaluate whether pGSN might diminish enhanced inflammatory response in human neutrophils during C. auris infection, secretion of inflammatory mediators was assessed. As shown in Fig. 4, we observed an increase in the secretion of interleukin-2 (IL-2), IL-4, IL-6, IL-8, and tumor necrosis factor alpha (TNF-α) by neutrophils under the influence of C. auris. When neutrophils before infection were preincubated with pGSN, a significant decrease in IL-4, IL-6, and TNF-α secretion was observed. Consistent with its immunomodulatory function, pGSN caused an increase in anti-inflammatory IL-10.

### Scavenger receptor class B is a target for pGSN on human neutrophils.

**FIG 5:**
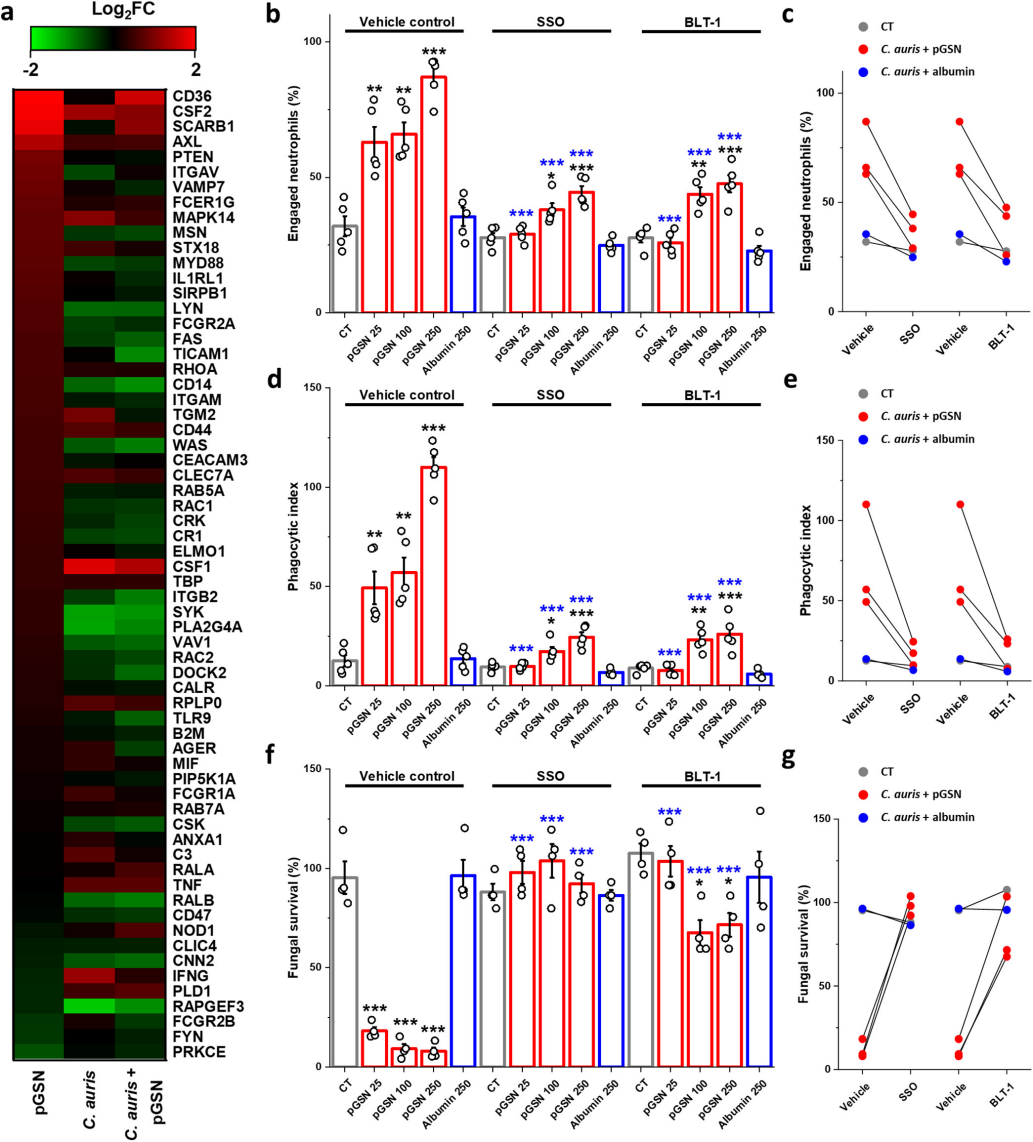
Plasma gelsolin enhances phagocytosis of C. auris through stimulation of the scavenger receptor class B type I (SR-BI). (a) Heat map of changes in gene expression of selected phagocytosis-related genes upon incubation with pGSN, C. auris cells, and preincubation with pGSN followed by addition of the yeast (*n* = 3). Results are presented as the log_2_ fold change (log_2_FC). (b to g) Effect of scavenger receptor class B type I (SR-BI) inhibitors SSO at 200 μM and BLT-1 at 1 μM compared to the vehicle control on fungal uptake (*n* = 5) (b and c), the phagocytic index (*n* = 5) (d and e), and yeast survival (*n* = 4) (f and g). Human neutrophils were preincubated with the indicated SR-BI inhibitors during serum starvation for 1 h; then, pGSN was added for 1 h, the cells were carefully washed, and C. auris was introduced to the neutrophils at an MOI of 1 for 2 h. (c, e, and g) Trend of the effect of SR-BI inhibitors compared to vehicle controls on fungal uptake, the phagocytic index, and fungal survival. Data are expressed as the mean ± SEM. *, *P* < 0.05; **, *P* < 0.01; ***, *P* < 0.001. Significance was determined by one-way ANOVA with Tukey’s test. The black asterisk refers to the comparison with the untreated condition within each group (vehicle or SSO- or BLT-1-treated cells), while the blue asterisk refers to the significance compared to the corresponding concentration in the vehicle control. Human albumin was used as a negative control.

The mechanism by which pGSN stimulates phagocytosis in human neutrophils is unknown. To shed light on the potential genes involved in the stimulation of phagocytosis by pGSN, we evaluated the expression of genes involved in this process. As shown in Fig. 5a, upon pGSN addition, the most significant increase was observed among genes from the scavenger receptor class B (SR-B) family, CD36 and SCARB1. Enhanced expression of CD36 was also confirmed using flow cytometry, as shown in Fig. S1 in the supplemental material. SR-B on immune cells is involved in processes of the innate immune response (13). To confirm that enhanced pGSN-mediated phagocytosis depends on increased SR-B expression, we inhibited these receptors using their antagonists, sulfosuccinimidyl oleate (SSO; CD36 inhibitor) and block lipid transport-1 (BLT-1; SR-B1 inhibitor) (36, 37). These inhibitors were added to neutrophils at the serum starvation stage and the preincubation with pGSN stage for 2 h. After incubation, the inhibitors and pGSN were washed off, and a medium with or without C. auris cells at an MOI of 1 was then added to the cells for 2 h. As shown in Fig. 5b to g, the SR-B inhibitors significantly decreased the beneficial effect of pGSN on all the aspects of neutrophil action, i.e., the number of engaged neutrophils (Fig. 5b and c), phagocytic index (Fig. 5d and e), and fungal survival (Fig. 5f to g). The SSO inhibitor entirely reversed the effects of pGSN on yeast survival; when SR-B was blocked during preincubation with pGSN, the fungal killing did not differ from untreated conditions (Fig. 5f). These results suggest that increased phagocytosis of C. auris by human neutrophils is mediated by pGSN’s ability to stimulate SR-B expression.

## DISCUSSION

Here, we investigated the immunomodulatory effects of pGSN on the neutrophil’s immune response to the rapidly emerging multidrug-resistant fungal pathogen C. auris, causing infections characterized by high mortality rates and high transmissibility in the hospital environment. Stimulation of human neutrophils with pGSN resulted in significantly increased phagocytosis efficiency and reduced the yeasts’ intracellular survival. Additionally, neutrophils preincubated with pGSN displayed a reduced ability to form NETs and to produce reactive oxygen species when activated with C. auris cells. Overall, a reduced proinflammatory response as a result of pGSN action was observed. The pGSN-enhanced ability of neutrophils to phagocytize fungi depends on SR-B, whose enhanced gene expression was observed upon pGSN stimulation and confirmed using specific inhibitors of these receptors. Since the innate immune response is a remarkably complex process, and immunocompromised patients often struggle with neutropenia, the number of circulating neutrophils may determine the efficiency of potential future administration of recombinant pGSN.

Recent studies indicate that C. auris cells can interact differently with immune cells depending on the strain, host cells, inoculum size, and incubation time. In their work, Wang et al. compared the ability to induce an immune response in a mouse model of infection with C. auris BJCA001 (clade I) and Candida albicans SC5314, pointing to C. auris as being less able to stimulate the immune system (28). Approximately 30% of bone marrow-derived macrophages (BMDMs) and nearly 0% of neutrophils were involved in phagocytosis of C. auris after 1 h of incubation at an MOI of 1. They also reported a low phagocytosis index and high fungal outgrowth after 6 and 24 h. Moreover, they recorded a slight increase in proinflammatory factors secreted by BMDMs, using an MOI of 5 and 6 h of incubation. Significantly fewer neutrophils were involved in phagocytosis in the work of Wang et al. Likely, the difference between the strains used in their study and those in our work and the mouse origin of the cells account for these differences. *N*-mannan, highly expressed in C. auris cells, masks exposure of the β-glucan, allowing fungal cells to evade innate immunity by restricting the access of dectin-1 to β-glucan. In human neutrophils, mannan-neutrophil interaction involves membrane receptors for mannose-linked proteins, which can behave as a major pathogen-associated molecular pattern (PAMP) to induce a cytokine response similarly as on human peripheral blood mononuclear cells (PBMCs) (38, 39). Despite this, the results are broadly consistent with our observations. Bruno et al. studied the immune response by host PBMCs mainly under the influence of clade I C. auris cells (29). They observed fungal macrophage uptake exceeding 50% after just 1 h of incubation, with an MOI of 3. Similarly, as in our work, they observed an increase in proinflammatory cytokines (IL-6 and TNF-α) under the influence of C. auris. In addition, they found that the increased production of cytokines depends on CLR complement receptors, which are not expressed on human neutrophils, suggesting the involvement of other signaling pathways in the induction of inflammatory processes, such as complement component C3, the increased expression of which was observed in our study, after the addition of C. auris cells (40). Despite using a different strain of C. auris and PBMCs, the number of cells involved in phagocytosis and the inflammatory response was similar to that demonstrated in our work. Johnson et al. showed in their work that C. auris B11203 (clade II), compared to C. albicans, avoids the immune response of human neutrophils and in the zebrafish model (19). Similarly, as in our study, C. auris at an MOI of 1 involved a small percentage of neutrophils, not limiting fungal outgrowth. At a 5-fold lower inoculum, C. auris did not affect the increased production of NETs, which seem to be inoculum dependent, with similar levels of reactive oxygen species production for the first 3 h of incubation.

Although pGSN has been studied for a relatively long time, the mechanism exploited by the protein to stimulate phagocytosis is not understood. In a mouse model of primary pneumococcal pneumonia, recombinant human pGSN (rhu-pGSN) caused enhanced bacterial clearance, reduced acute inflammation, and improved survival. *In vitro*, rhu-pGSN rapidly improved lung macrophage uptake and killing of bacteria (Streptococcus pneumoniae, Escherichia coli, and Francisella tularensis) (41). pGSN triggers bactericidal functions in lung macrophages by activating the phosphorylation of macrophage nitric oxide synthase type III (NOS3). rhu-pGSN failed to enhance bacterial killing by NOS3-lacking macrophages *in vitro* or bacterial clearance in NOS3-knockout mice *in vivo*. In other studies, preincubation of macrophages with pGSN prior to adding P. aeruginosa cells resulted in a dose-dependent increase in the level of phagocytosing cells (11). This effect, similar to the observation in our study, was not so prominently observed when macrophages were treated simultaneously with both pGSN and bacteria. In our work, we observed that enhanced expression of SR-B after pGSN treatment caused significant improvement in phagocytic uptake and the killing of C. auris cells by human neutrophils. Recent studies demonstrated that SR-B receptors mediate the host defense against Cryptococcus neoformans and C. albicans in human macrophages by direct binding to cell wall β-glucan. The accelerated mortality rate in CD36-deficient mice correlated with the higher number of yeast cells in the spleen and liver during C. neoformans infection (13). Macrophages lacking CD36 demonstrate reduced internalization of S. aureus and its component lipoteichoic acid (LTA), accompanied by a marked defect in TNF-α and IL-12 production. As a result, CD36-knockout mice fail to efficiently clear S. aureus *in vivo*, resulting in profound bacteremia (42). TLR4 and TLR9 ligands can bind to SR-B1, which acts as a scavenger, facilitating the TLR ligands’ bioavailability and consequently limiting the immune response (43). CD36 downregulation decreases the phagocytic ability of macrophages in the peritoneum of women with endometriosis (44). SR-B1-deficient mice struggle with enhanced production of proinflammatory cytokines, autoimmunity, and impairment in phagocytic killing (45). However, some SR-B1 ligands, such as serum amyloid A (SAA), glycated or oxidized Apo A-I, or dysfunctional HDLs, have been shown to promote inflammation (46–48). Thus, SR-B1 plays a dual role in inflammation and may represent a novel target for designing new immunotherapies. Collectively, these data demonstrate that the SR-B are nonredundant components of an evolutionarily conserved pathway for fungal recognition and innate immunity necessary for controlling fungal infections, both *in vitro* and *in vivo.*

In our study, we show for the first time that pGSN exhibits an immunomodulatory role in the response of human neutrophils during fungal infections and that the anti-inflammatory effects and enhancement of phagocytosis depend on pGSN-mediated SR-B upregulation. These results suggest that pGSN might serve as a single or adjuvant therapy in immunocompromised patients struggling with fungal infections, due to its immunomodulatory effect on human neutrophils, demonstrated by increased phagocytosis, with a simultaneous attenuation of the inflammatory response.

## MATERIALS AND METHODS

### Plasma gelsolin.

The recombinant human plasma gelsolin used in our study was expressed in E. coli cells and provided by BioAegis Therapeutics (North Brunswick, USA).

### Fungal preparations.

This study used a strain first isolated in Japan (clade II), Candida auris 21092 (DSMZ, Braunschweig, Germany). The strain was obtained from glycerol stocks stored at −80°C, plated on Sabouraud dextrose with chloramphenicol agar (Lab-Agar; Biomaxima, Lublin, Poland), and routinely grown at 30°C. Hemocytometer counts were used to estimate the number of yeast cells. Exponentially growing *Candida* cells were suspended and diluted at the required cell number in cell culture media for coculture with human neutrophils.

### Neutrophil isolation and culture.

Blood was collected from healthy donors under the approval of the Bioethics Committee at the Medical University of Bialystok (APK.002.234.2021). Neutrophils were isolated by density gradient centrifugation using PolymorphPrep (Progen, Heidelberg, Germany). Cells were further counted on a hemocytometer and suspended in antimycotic and serum-free RPMI 1640 medium (ATCC, Manassas, USA) to prevent the possible influence of plasma gelsolin remaining in fetal bovine serum (FBS). Cells were incubated at 37°C with 5% CO_2_.

### Uptake of zymosan pHrodo particles.

A phagocytosis assay was used to determine the impact of preincubation and the simultaneous addition of pGSN on pHrodo zymosan bioparticle (Sartorius AG, Göttingen, Germany) uptake by human neutrophils. Neutrophils (4 × 10^4^ cells/well in a 96-well plate) were serum starved for 1 h and either preincubated with pGSN or left in a serum-free medium for 1 h. Then, the cells were carefully washed with phosphate-buffered saline (PBS), and 5 μg of zymosan particles dissolved in RPMI 1640 medium were added to the neutrophils without pGSN (preincubation) and with pGSN (no preincubation) for 2 h of incubation in the IncuCyte SX1 platform, housed inside a cell incubator at 37°C and 5% CO_2_. Two images per well from four replicates were taken using 20× magnification. The number of internalized zymosan particles was analyzed using the IncuCyte basic software. The results were compared to the untreated control, normalized to 1.0, and presented as a fold change in the uptake of zymosan particles.

### Neutrophil engagement and phagocytosis.

Briefly, live, calcofluor white (Sigma-Aldrich, St. Louis, USA)-stained (0.25 μg/mL for 30 min; room temperature [RT]) C. auris cells at MOIs of 1 and 5 were added for 2 h to 1 × 10^5^ neutrophils seeded onto 0.01% poly-l-lysine (Sigma-Aldrich)-treated sterilized glass coverslips. Before adding yeasts, neutrophils were serum starved (1 h), preincubated with pGSN (1 h), and washed with PBS. After 2 h of infection, the coverslips were washed with PBS and fixed in 3.7% paraformaldehyde (PFA) for 15 min at RT. After permeabilization (0.1% Triton X-100; 10 min; RT) and blocking (0.1% bovine serum albumin; 30 min; RT), the cells were washed and stained with phalloidin-Texas red (Thermo Fisher Scientific, Waltham, USA) at a dilution of 1:40 for 1 h, at RT, in the dark. The coverslips were mounted with antifade fluorescence mounting medium (Abcam, Cambridge, UK) and examined by confocal microscopy. The results are presented as the engaged cells (the percentage of cells uptaking or adherent to fungal cells) and phagocytic index (the total number of fungal cells taken up per 100 cells). Data were obtained from 5 separate experiments by analyzing at least 500 neutrophils per coverslip.

### Fungal survival assay.

Serum-starved (1 h) neutrophils (1 × 10^6^ cells/well in a 24-well plate) were preincubated with pGSN (1 h), washed, and then infected with C. auris cells at MOIs of 1 and 5 for 2 h. The cultures were collected using cell scrapers and transferred to Eppendorf tubes. The neutrophils were lysed by sonication for 10 min. Serial dilutions were plated on Sabouraud agar for yeast outgrowth assessment and left for 48 h of incubation at 30°C.

### NETosis assay.

To assess the formation of neutrophil extracellular traps (NETs), 1 × 10^5^ cells were seeded onto 0.01% poly-l-lysine (Sigma-Aldrich) functionalized sterilized glass coverslips. Neutrophils were serum starved (1 h), preincubated with pGSN (1 h), and infected with C. auris (MOI = 5) for 2 h at 37°C and 5% CO_2_. A condition with neutrophils treated with phorbol 12-myristate 13-acetate (PMA; Cayman Chemicals, Ann Arbor, USA) at 100 nM was also included. After incubation, the cells were washed with PBS, fixed in 3.7% PFA, permeabilized with 0.1% Triton X-100, and blocked in 0.1% bovine serum albumin, as mentioned above. Next, the cells were stained with mouse anti-myeloperoxidase (MPO) antibody (catalog number MA1-80878; Sigma-Aldrich) at a dilution of 1:500 for 1 h at RT. The cells were stained with a secondary anti-mouse antibody conjugated with Alexa Fluor 488 dye (catalog number ab150113; Abcam, Cambridge, UK) at a dilution of 1:1,000 for 1 h at RT, in the dark. The cell nuclei were counterstained with DAPI (4′,6-diamidino-2-phenylindole). The coverslips were mounted with antifade fluorescence mounting medium (Abcam) and examined by confocal microscopy. The number of cells producing NETs was manually counted. Five images per coverslip were taken randomly and analyzed (*n* = 4).

In a parallel experiment, the impact of preincubation and the simultaneous addition of pGSN during C. auris infection (MOI = 5) on extracellular DNA (eDNA) release was determined using the IncuCyte instrument (Sartorius). Briefly, neutrophils (4 × 10^4^ cells/well in a 96-well plate) were prepared and infected, as previously mentioned. Simultaneously to the addition of fungal cells, Cytotox red dye (Sartorius) at a final concentration of 250 nM was introduced to the neutrophils and left for 2 h of incubation at 37°C and 5% CO_2_. Two images per well were taken from four technical replicates using 20× magnification, and then the eDNA area was analyzed using the IncuCyte basic software. The results were compared to the untreated control, normalized to 1.0, and presented as a fold change in the eDNA area.

### Reactive oxygen species production.

To assess the generation of reactive oxygen species (ROS), neutrophils were stained with DCFH-DA (2′-7′-dichlorofluorescin diacetate; Sigma-Aldrich) in PBS for 10 min in the dark, washed with PBS, and plated at 2 × 10^5^ cells/well in a 96-well plate. The cells were serum starved for 1 h and preincubated with pGSN for 1 h; C. auris cells at an MOI of 5 were then introduced for 2 h. A PMA (100 nM) control was also included. The fluorescence (488/535 nm) was recorded on the Varioskan Lux microplate reader (Thermo Fisher Scientific). The background fluorescence was determined for each condition and subtracted from the total fluorescence values before data analysis. The results were compared to the untreated control, normalized to 1.0, and presented as a fold change in the ROS production.

### Quantification of cytokine and chemokine secretion.

Secretion of IL-2, IL-6, IL-8, gamma interferon (IFN-γ), TNF-α, and granulocyte-macrophage colony-stimulating factor (GM-CSF) was assessed using the Bio-Plex Pro human cytokine assay (Bio-Rad Laboratories, Hercules, USA). Neutrophil cells (1 × 10^6^/well) were cultured on 24-well culture plates. The cells were serum starved (1 h), preincubated with pGSN (1 h), and/or infected with C. auris yeast at an MOI of 5. Then, supernatants were collected, centrifuged to eliminate residual cells, and subjected to cytokine secretion assessment on the Bio-Plex 200 system (Bio-Rad).

### Western blot analysis.

Human neutrophils were serum starved, preincubated with pGSN, and infected with C. auris at an MOI of 5, as previously mentioned. The whole-cell lysate from 1 × 10^7^ neutrophils was prepared using radioimmunoprecipitation assay (RIPA) buffer, supplemented with protease inhibitors (Sigma-Aldrich). The Bradford assay (Bio-Rad) was performed to determine the protein concentration. Lysates were subjected to electrophoresis using 10% sodium dodecyl sulfate-polyacrylamide (SDS-PAGE) at a concentration of 10 μg protein per lane. After SDS-PAGE separation, proteins were blotted onto polyvinylidene fluoride (PVDF) membranes. Next, the membranes were submerged in methanol and blocked for 1 h in 5% nonfat dry milk in TBS-T (150 mM NaCl, 50 mM Tris base, 0.05% Tween 20, pH = 7.4). The blocked protein blots were incubated with rabbit anti-NOX2/gp91phox (dilution, 1:300; catalog number BS-3889R; Thermo Fisher), anti-neutrophil elastase (dilution, 1:1,000; catalog number ab21595; Abcam), anti-TLR4 (dilution, 1:1,000; catalog number ab13556; Abcam), and mouse anti-PAD4 (dilution, 1:2,000; catalog number ab128086; Abcam), anti-Cit H3 (dilution, 1:1,000; catalog number ab10799; Abcam), and anti-β-actin (dilution, 1:5,000; catalog number A5441; Sigma-Aldrich), in TBS-T at 4°C overnight, followed by incubation with goat anti-rabbit IRDye 800CW IgG (catalog number ab216773; Abcam) and goat anti-mouse IRDye 800CW IgG (catalog number 926-32210; LiCor Biosciences, Lincoln, USA) secondary antibody in TBS-T (dilution, 1:10,000) at room temperature for 1 h, in the dark. The protein blots were visualized using the Odyssey imaging system (LiCor Biosciences). Band intensities were quantified using Image Studio Acquisition software. Data are presented as the relative band intensity of the protein of interest compared to the untreated samples and normalized to β-actin.

### Expression of phagocytosis-related genes.

Total RNA was extracted using the universal RNA purification kit (catalog number E3598-02; EURx, Gdansk, Poland) from 1 × 10^7^ neutrophils per well seeded in 6-well cell culture plates. Cells were serum starved (1 h), preincubated with pGSN (1 h), and infected or not with C. auris yeast at an MOI of 5 for 2 h. The cells were scratched, transferred to Eppendorf tubes, and centrifuged, and the supernatant was discarded. The concentration and purity of the isolated RNA were evaluated using a Qubit 4 fluorometer (Thermo Fisher Scientific). cDNA was synthesized using the iScript cDNA synthesis kit (catalog number 1708891; Bio-Rad). Reverse transcription-quantitative PCR (qRT-PCR) was performed with 20 ng of cDNA in a 20-μL reaction mixture containing SsoAdvanced universal SYBR green supermix (catalog number 1725274; Bio-Rad) using phagocytosis PrimePCR plates (catalog number 10047255; Bio-Rad) on the CFX Connect real-time PCR detection system (Bio-Rad) with the following amplification program: 2 min at 95°C, followed by 40 cycles of 5 s at 95°C and 30 s at 60°C. GAPDH (glyceraldehyde-3-phosphate dehydrogenase) was used as an internal control. The gene expression levels were reported as the relative quantity, expressed using the comparative cycle threshold (*C_T_*) method (2^-ΔΔCt^), and presented as log_2_FC. The raw *C_T_* values from the qPCR experiment are presented in Data Set S1 in the supplemental material.

### SR-B inhibition assay.

To determine whether SR-B antagonists inhibit the stimulation of phagocytosis and fungal killing in human neutrophils triggered by pGSN, we used sulfosuccinimidyl oleate (SSO; catalog number ab145039; Abcam) at 200 μM and block lipid transport-1 (BLT-1; catalog number SML0059; Sigma-Aldrich) at 1 μM. Briefly, inhibitors were introduced for a total of 2 h to the cells during serum starvation (1 h) and pGSN preincubation (1 h) and then carefully washed with PBS and infected with C. auris at an MOI of 1. Assessment of the fungal uptake, phagocytic index, and fungal survival was performed as mentioned above.

### Statistical analysis.

The statistical parameters, including the exact number of replicates and statistical significance, are reported in the figures and figure legends. The statistical tests were performed using OriginPro 2021 software (build 9.8.0.200; OriginLab Corporation, Northampton, USA). Specific statistical tests are indicated in the figure legends.

### Data availability.

All data needed to evaluate the conclusions in the paper are present in the paper. Additional data related to this paper may be requested from the authors.
