# Supplementary material for: Plasma Gelsolin Enhances Phagocytosis of Candida auris by Human Neutrophils through Scavenger Receptor Class B
Source: Microbiol Spectr. 2023 Feb 21;11(2):e04082-22. doi: 10.1128/spectrum.04082-22 (PMC10101141; doi:10.1128/spectrum.04082-22)
Supplement: Supplemental file 2 — Fig. S1. Download spectrum.04082-22-s0002.pdf, PDF file, 0.3 MB [file spectrum.04082-22-s0002.pdf]

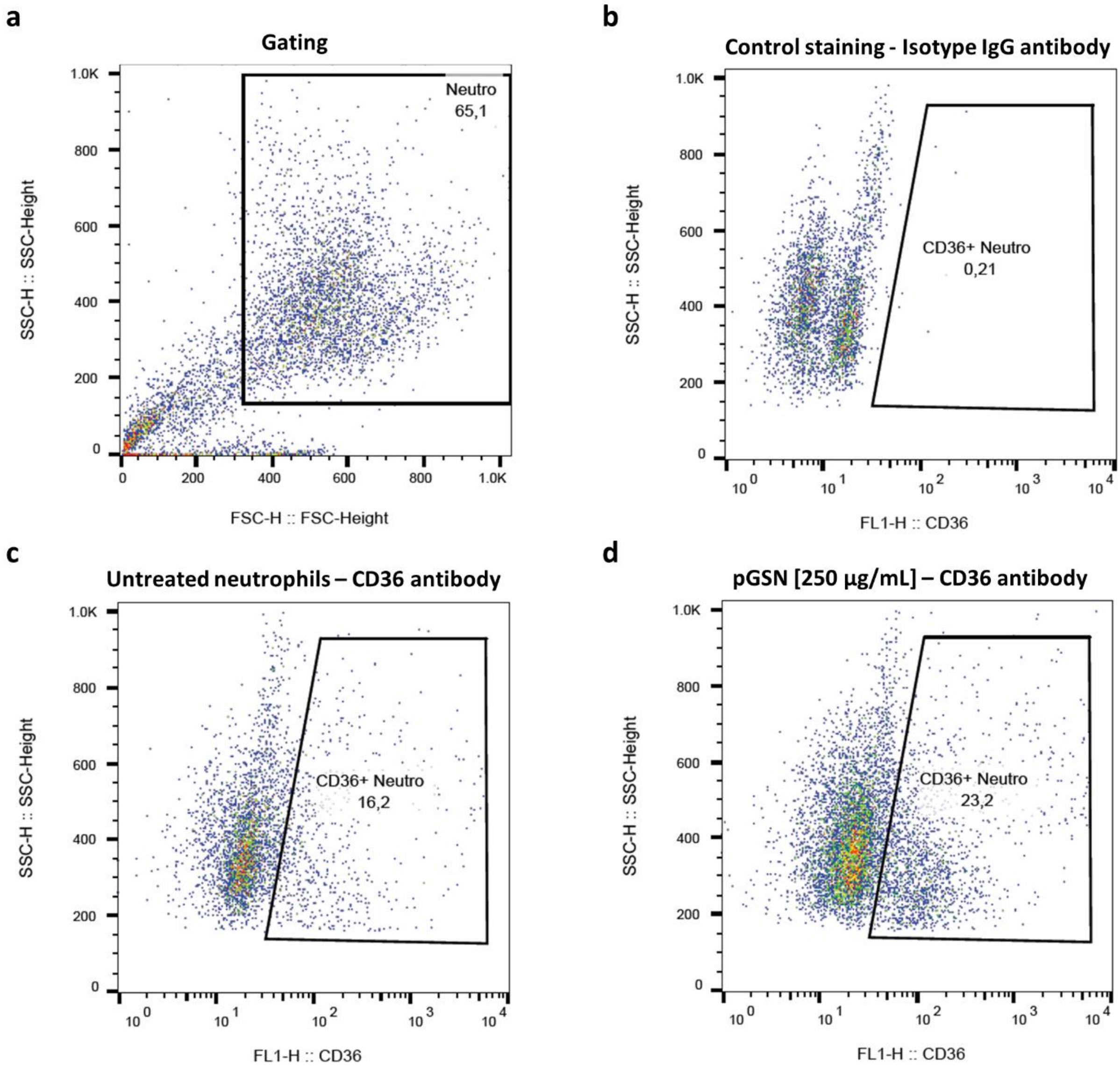

Figure S1. Flow cytometry analysis of CD36 expression in human neutrophils. Neutrophils were serum starved for 1h, then either incubated or not with 250 µg/mL of pGSN for 1h. Subsequently, cells were fixed with 2% paraformaldehyde for 30 min and stained with FITC Mouse Anti-Human IgG (#555786, BD Biosciences, Franklin Lakes, NJ, U.S.) or FITC Mouse Anti-Human CD36 (#656152, BD Biosciences). After staining, cells were immediately subjected to a flow cytometer analysis on the FACSCalibur flow cytometry system (BD Biosciences). **a)** Gating of neutrophils in the sample. **b)** Isotype control staining. **c)** Expression of CD36 in untreated and **d)** pGSN-treated neutrophils. Data were analyzed using FlowJo software version 10.6.1 (Tree Star, Inc., Ashland, OR, U.S.).
